# Feasibility of large language models for CEUS LI-RADS categorization of small liver nodules in patients at risk for hepatocellular carcinoma

**DOI:** 10.3389/fonc.2024.1513608

**Published:** 2024-12-18

**Authors:** Jiayan Huang, Rui Yang, Xiaotong Huang, Keyu Zeng, Yan Liu, Jun Luo, Andrej Lyshchik, Qiang Lu

**Affiliations:** ^1^ West China Hospital of Sichuan University, Chengdu, China; ^2^ Department of Ultrasound, Affiliated Hospital of Panzhihua University, Panzhihua, China; ^3^ Department of Ultrasound, Sichuan Academy of Medical Sciences and Sichuan Provincial People’s Hospital, Chengdu, China; ^4^ Thomas Jefferson University Hospital , Jefferson University Hospitals, Philadelphia, PA, United States

**Keywords:** hepatocellular carcinoma (HCC), large language model (LLM), diagnosis, CEUS (Contrast-enhanced ultrasound), ultrasound

## Abstract

**Background:**

Large language models (LLMs) offer opportunities to enhance radiological applications, but their performance in handling complex tasks remains insufficiently investigated

**Purpose:**

To evaluate the performance of LLMs integrated with Contrast-enhanced Ultrasound Liver Imaging Reporting and Data System (CEUS LI-RADS) in diagnosing small (≤20mm) hepatocellular carcinoma (sHCC) in high-risk patients.

**Materials and Methods:**

From November 2014 to December 2023, high-risk HCC patients with untreated small (≤20mm) focal liver lesions (sFLLs), were included in this retrospective study. ChatGPT-4.0, ChatGPT-4o, ChatGPT-4o mini, and Google Gemini were integrated with imaging features from structured CEUS LI-RADS reports to assess their diagnostic performance for sHCC. The diagnostic efficacy of LLMs for small HCC were compared using McNemar test.

**Results:**

The final population consisted of 403 high-risk patients (52 years ± 11, 323 men). ChatGPT-4.0 and ChatGPT-4o demonstrated substantial to almost perfect intra-agreement for CEUS LI-RADS categorization (κ values: 0.76-1.0 and 0.7-0.94, respectively), outperforming ChatGPT-4o mini (κ values: 0.51-0.72) and Google Gemini (κ values: -0.04-0.47). ChatGPT-4.0 had higher sensitivity in detecting sHCC than ChatGPT-4o (83%-89% vs. 70%-78%, *p* < 0.02) with comparable specificity (76%-90% vs. 83%-86%, *p* > 0.05). Compared to human readers, ChatGPT-4.0 showed superior sensitivity (83%-89% vs. 63%-78%, *p* < 0.004) and comparable specificity (76%-90% vs. 90%-95%, *p* > 0.05) in diagnosing sHCC.

**Conclusion:**

LLM integrated with CEUS LI-RADS offers potential tool in diagnosing sHCC for high-risk patients. ChatGPT-4.0 demonstrated satisfactory consistency in CEUS LI-RADS categorization, offering higher sensitivity in diagnosing sHCC while maintaining comparable specificity to that of human readers.

## Introduction

Liver cancer is the sixth most common cancer and the third leading cause of cancer-related deaths worldwide, with over 830,000 deaths in 2020 and rising mortality rates (1). Among all the pathological subtypes of liver cancer, hepatocellular carcinoma (HCC) accounts for the majority of cases. However, due to the complex dual blood supply to liver and the multistage process of HCC, radiological diagnosis of HCC remains challenging (2). Notably, the early diagnosis of HCC, especially for tumors measuring 2 cm or smaller in diameter (small HCC), due to it offers more treatment options, reduced risk of complications and better prognosis (3).

The Contrast-enhanced Ultrasound Liver Imaging Reporting and Data System (CEUS LI-RADS) released by American College of Radiology (ACR)aims to improve the accuracy and consistency of HCC diagnosis in patients at high-risk (4, 5). The implementation of structured reporting in radiology plays a pivotal role in improving communication, fostering collaboration among medical practitioners, and standardizing reporting language across institutions (6). Moreover, the characterization of focal liver lesions (FLLs) using CEUS LI-RADS, based on imaging features derived from B-mode and multiphasic CEUS enhancement patterns, establishes a robust foundation for the application of artificial intelligence (AI) in imaging diagnosis. Large Language Models (LLM) represent a specialized AI application that focuses on comprehending and generating text resembling human-like language. Recently conducted research on the interaction strategy between humans and LLM has shown promising results in terms of LLM-agreement and diagnostic accuracy for predicting benign and malignant thyroid nodules using the ACR Thyroid Imaging Reporting and Data System (TI-RADS) (7).

Amid significant advancements in LLMs, AI chatbots like ChatGPT have gained increasing attention across various fields (8). The Chatbot (primarily ChatGPT 4.0) has showed potential in transforming unstructured free-text reports into organized formats (6, 9). However, the impressive capability of LLMs in rapid and standardized language processing notwithstanding, concerns have increasingly arisen regarding the assessment of their agreement and accuracy in generating prompt output. The subjective question-answering format may inadvertently be influenced by existing biases and disparities, thereby overlooking crucial aspects of transparency and accuracy when investigating the applications of LLMs (7, 10). Notably, LLMs such as ChatGPT 4.0 do not support concurrent recognition or processing of multiple images. Given that this approach is more practical for handling natural language, the medical application of LLMs, particularly in processing radiology reports, represents a critical and cutting-edge area of research at the forefront of LLM advancements (6–9, 11). Despite the promising efficacy of LLMs demonstrated by a rapidly emerging plethora of studies, their potential in aiding real-world clinical applications remains controversial.

To our knowledge, the performance of LLMs in classifying FLLs based on CEUS LI-RADS reports, particularly regarding their output LLMs-agreement and accuracy, has not been previously reported. Thus, the purpose of our study was to evaluate the intra- and inter-agreement of four publicly available LLMs (Google Gemini, ChatGPT-4.0, ChatGPT-4o, and ChatGPT-4o mini) in CEUS LI-RADS categorization using structured CEUS reports from patients with small FLLs. Moreover, the diagnostic accuracy of LLMs in diagnosing small HCC was also investigated, using a composite reference standard as previously described (12).

## Materials and methods

This retrospective study was approved by the ethics committee of West China Hospital of Sichuan University, and written informed consent was waived. To consecutively collect participants, 172 samples in this study were drawn from our previous investigation (12). Ultrasound images of these patients were used to develop structured reports for LLMs, while in the earlier research, they were used for evaluating the diagnostic accuracy of CEUS LI-RADS (version 2017).

### Study design

**Figure 1:**
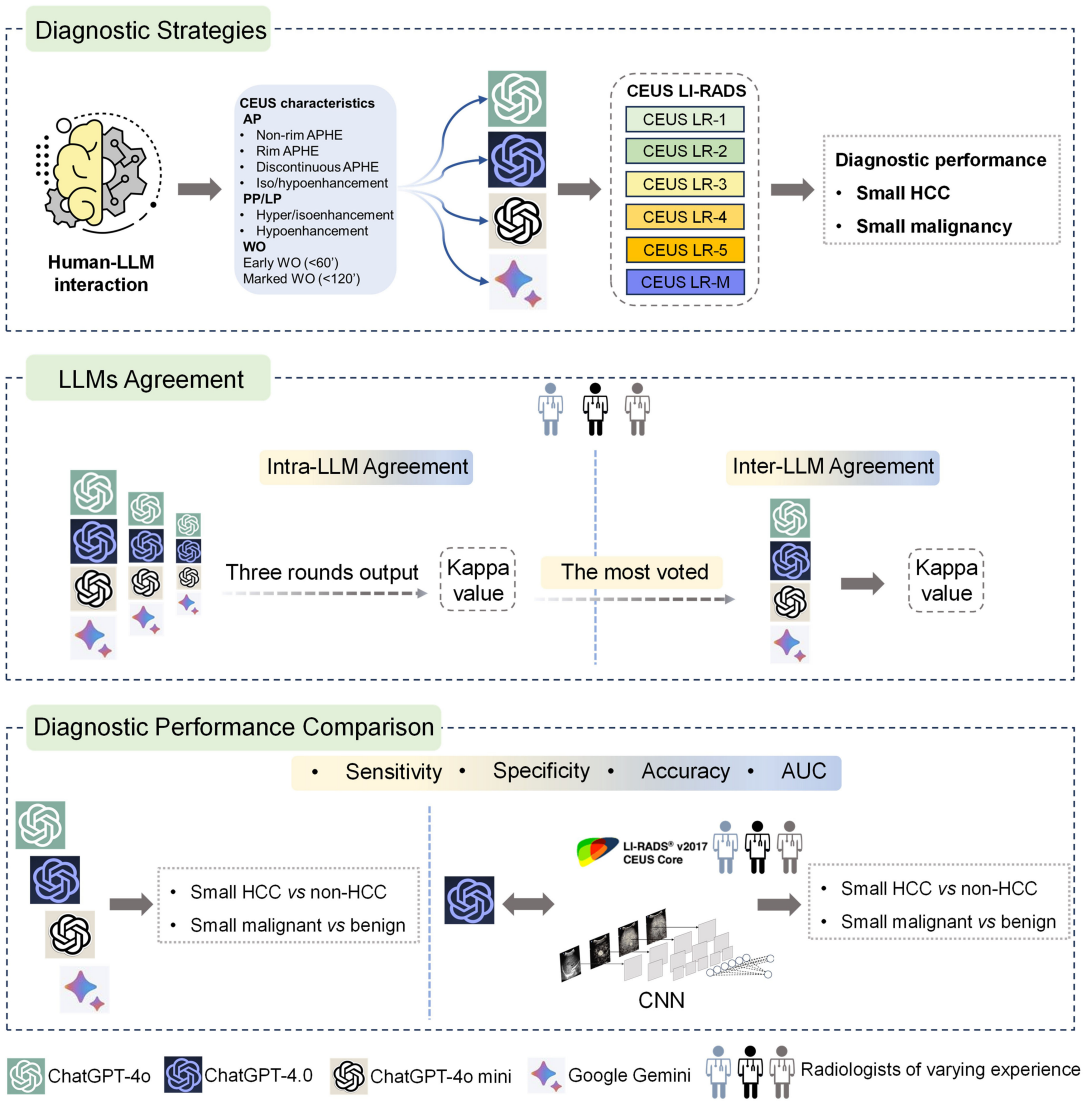
Graphical representation of study design. Overall, LLMs were integrated with structured reports based on the CEUS LI-RADS for diagnosing sHCC (the top box). First, the LLMs' agreement was evaluated by comparing intra-LLM consistency, with the most frequently voted category used for further inter-LLMs agreement assessment (the middle box). Second, the diagnostic performance of the LLMs was assessed in comparison to human readers utilizing CEUS LI-RADS (Ver. 2017) for diagnosing sHCC, as well as compared to a CNN model (the bottom box). LLMs, large language models; CEUS LI-RADS, Contrast-enhanced Ultrasound Liver Imaging Reporting and Data System; sHCC, small hepatocellular carcinoma; CNN, convolutional neural network.

This study investigated four publicly available LLM chatbots, including three versions of ChatGPT (ChatGPT-4.0, ChatGPT-4o, ChatGPT-4o mini) and Google Gemini. Ultrasound images of small focal liver lesions (sFLLs) were assessed by six certified ultrasound radiologists with 3 to 20 years of liver CEUS experience using CEUS LI-RADS (Version 2017). Structured reports were then rendered and entered into LLMs for prompt CEUS LI-RADS categorization. Three output rounds (with an extra round if completely inconsistent) were performed to evaluate the intra-agreement of LLM in sFLLs categorization, with a majority vote deciding the final CEUS LI-RADS category. To avoid space-time impact, outputs were spaced three days apart. The diagnostic efficacy of LLMs for sHCC was compared by analyzing reports from readers with varying expertise. Furthermore, the best-performing LLM was compared to human readers and a convolutional neural network (CNN) model in diagnosing sHCC (**Figure 1**).

### Patient selection and reference standard

Consecutive patients underwent hepatic CEUS examinations were retrospectively collected from November 2014 to December 2023. The inclusion criteria were: *(a)* aged 18 or older; *(b)* HCC risk factors involving cirrhosis or chronic hepatitis B (HBV); *(c)* untreated hepatic nodules ≤20 mm on imaging (ultrasound, CT or MRI); *(d)* less than two sFLLs, with the larger tumor selected for analysis to minimize the impact of multiple injections on contrast enhancement. Exclusion criteria included: *(a)* indeterminate pathology, contrast-enhanced CT/MRI results, or incomplete follow-up; *(b)* poor-quality ultrasound images. HCC risk factors were defined as any cause of cirrhosis and/or HBV, per the American Association for the Study of Liver Diseases guidelines (13). Patients with a history of HCC treatment were excluded to minimize the influence from post-treatment changes.

This study used a composite reference standard as previously described in our earlier investigation (12). In brief, all lesions were diagnosed by histopathology, while contrast-enhanced CT/MRI were used for LR-1 or LR-5 nodules. Diagnosis for LR-2, LR-3, and LR-4 nodules involved imaging follow-up (≥12 months), pathology, or multidisciplinary recommendations, while LR-M lesions were diagnosed by histopathology. The processing of the CEUS examination is detailed in **Supplementary Material Appendix S1**.

### CEUS LI-RADS category assignment by LLMs

After independent review of the images by radiologists, a separate radiologist translated the structured reports, including patients’ clinical and ultrasound characteristics, from Chinese to English. Then, the reports were input into ChatGPT-4o mini (14), ChatGPT-4o (15), ChatGPT-4.0 (16) and Google Gemini (17) for prompt CEUS LI-RADS classification. LLMs were integrated with CEUS LI-RADS reports to evaluate their agreement and diagnostic performance in diagnosing sHCC, since LLMs currently cannot interpret multiple images directly. The CEUS LI-RADS classification process for human readers and LLMs is detailed in **Supplementary Material Appendix S2** and **Supplementary Figure S1**.

### End-to-end CNN model of small FLLs

End-to-end CNN models involving baseline and multiphase CEUS images were developed for sHCC diagnosis. Patients were randomly divided into training (59.8%, 241 of 403) and validation sets (40.2%, 162 of 403). The diagnostic efficacy of the CNN for sHCC was evaluated using the validation cohort. Algorithm and network structures were established as described in previous investigations (7, 18). Due to the characterization of FLLs according to CEUS LI-RADS, which incorporates imaging features from both baseline and multiphasic CEUS images, results were derived by integrating outputs from each modality using a weighted averaging method. The CNN was developed using ultrasound images of sFLLs, with tumor segmentation performed by a radiologist with three years of liver CEUS experience, utilizing MITK Workbench (https://docs.mitk.org/nightly/index.html). The model was built and executed in Python (version 3.10.3; Python Software Foundation, Wilmington, Delaware, USA). Detailed information regarding network structures and parameters is provided in **Supplementary Material Appendix S3**, **Supplementary Figure S2** and **Supplementary Table S1**.

### Statistical analysis

Fleiss’ Kappa and Cohen’s Kappa tests were used to assess intra- and inter-LLM agreements, respectively. A best-of-three strategy was used to identify the preferred LLM category for sFLLs. Agreement strength was classified using the Landis and Koch scale: 0-0.20 as poor; 0.21-0.40 as fair; 0.41-0.60 as moderate; 0.61-0.80 as substantial; and 0.81-1.00 as almost perfect.

LR-5 category is designated for predicting HCC according to CEUS LI-RADS, whereas LR-4, LR-5, and LR-M are categorized as indicative of malignancy (19). The diagnostic performance of LLMs, human readers, and CNN strategy for sHCC was assessed by calculating sensitivity, specificity, accuracy and area under the receiver operating characteristic curve (AUC) based on standard procedures (12). Sensitivity, specificity, and accuracy were compared among LLMs, between LLMs and human readers, and between LLMs and CNNs using the McNemar test. AUCs for sHCC were compared among LLMs, human readers, and CNN using DeLong test.

Statistical analyses were conducted using R packages (R 4.1.2 [Puppy Cup], The R Foundation, Vienna, Austria) and MedCalc software (MedCalc22.030, Ostend, Belgium). A P-value less than.05 indicated statistical significance.

## Results

### Patients and liver nodule characteristics

**Figure 2:**
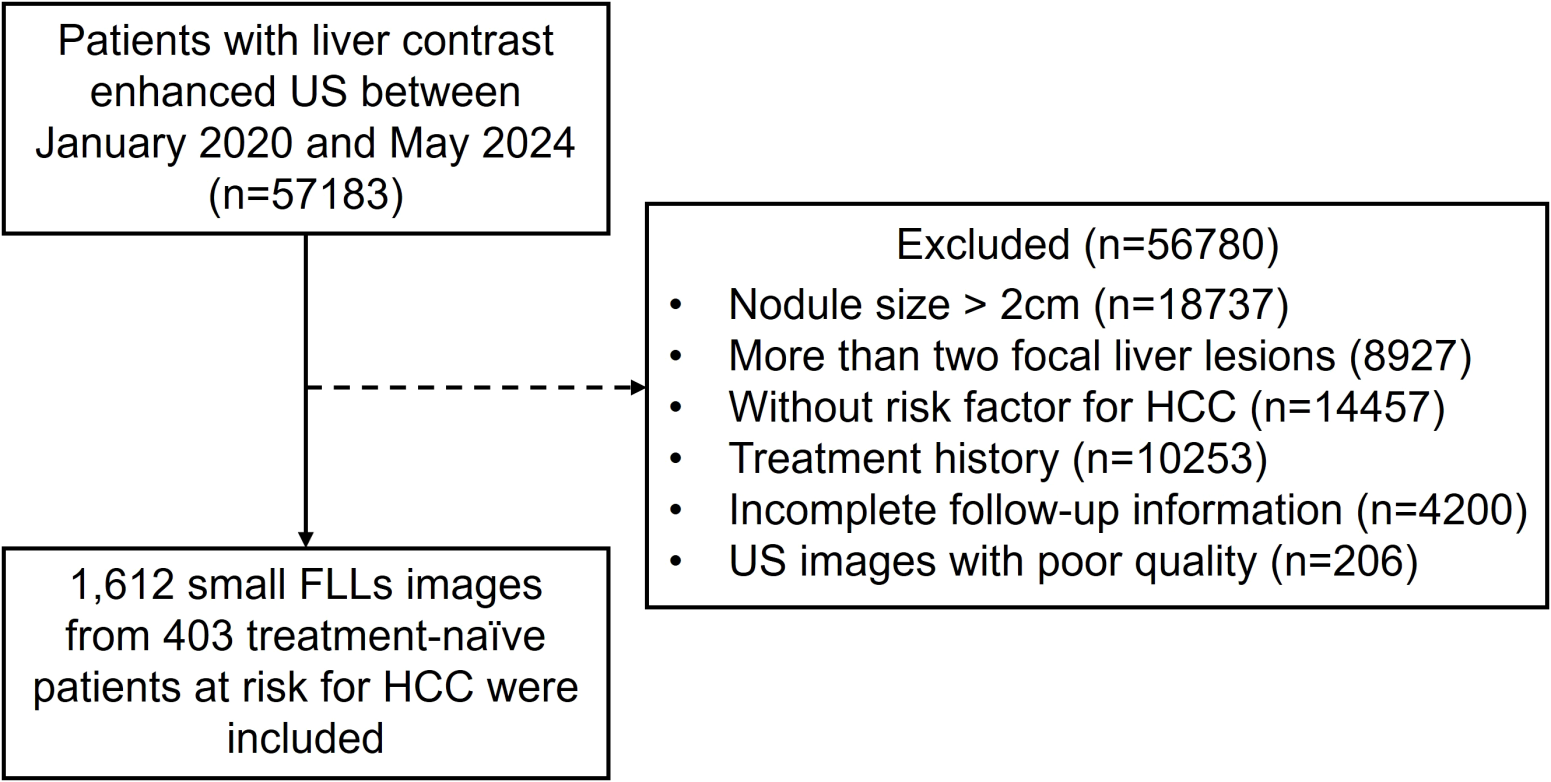
Study population flowchart. US, ultrasound; HCC, hepatocellular carcinoma; FLL, focal liver lesion.

**Figure 3:**
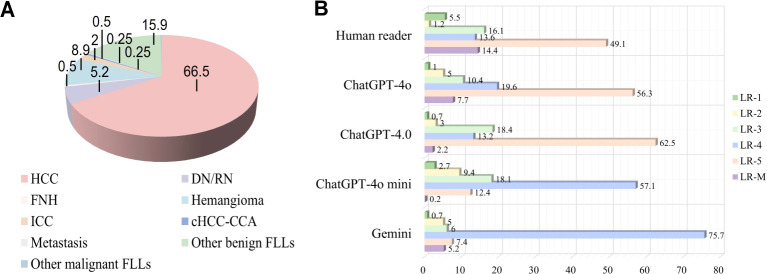
Pathological composition and CEUS LI-RADS categories of liver nodules classified by human reader and LLMs. **(A)** Pie chart depicts the pathological composition of 403 small FLLs according to the reference standard. **(B)** Horizontal stacked bar chart illustrates the distribution of CEUS LI-RADS categories for small FLLs as assigned by both LLMs and human readers using CEUS LI-RADS category. CEUS LI-RADS, Contrast-enhanced Ultrasound Liver Imaging Reporting and Data System; FLL, focal liver lesions; LLMs, large language models; HCC, hepatocellular carcinoma; FNH, focal nodular hyperplasia; ICC, intrahepatic cholangiocarcinoma; DN, dysplastic Nodule; RN, regenerative Nodule; cHCC-ICC, combined hepatocellular-cholangiocarcinoma.

A total of 1612 representative ultrasound images, including B-mode, arterial phase, portal phase, and late phase images (one representative image per phase), were obtained from 403 patients at risks of HCC with sFLLs (**Figure 2**). Of the 403 patients (mean age, 52.3 years ± 10.8; age range, 21–81 years), 323 (80.1%) were men. The mean size of sFLLs was 16.1 mm ± 3.4. Clinical features of patients involving age, gender, liver disease etiology, nodule size, and pathological results are exhibited in **Table 1**. Based on the composite reference standard, 263 liver nodules were proved by pathology, 65 by follow-up, and 75 by contrast enhanced CT or MRI (including 42 HCC and 33 hemangioma). The median follow-up period was 15.2 months (range 12–41 months). The constitution of 403 sFLLs and the distribution of CEUS LI-RADS categories, as determined by human readers and LMMs, are depicted in **Figure 3**.

**Table 1:** Clinicopathological characteristics of patients.

Characteristic	Value
Sex	
Men	323 (80.1)
Woman	80 (19.9)
Mean age (y)^*^	52.3 ± 10.8 (21–81)
Mean nodule size (mm)^*^	16.2 ± 3.4 (0.7–2)
Liver disease etiologic cause	
HBV	374 (92.8)
HCV	11 (2.7)
HBV and HCV	6 (1.5)
PBC	1 (0.2)
Alcohol	4 (1)
Unknown etiology	7 (1.7)
Cirrhosis	162 (40.2)
Pathologic Analysis	
HCC	223 (55.3)
Well differentiated	5 (1.2)
Moderately differentiated	161 (40)
Poorly differentiated	57 (14.1)
DN/RN	21 (5.2)
FNH	2 (0.5)
Hemangioma	3 (0.7)
ICC	8 (2)
cHCC-CCA	2 (0.5)
Metastasis	1 (0.2)
Reactive lymphoid hyperplasia	1 (0.2)
Biliary adenoma	1 (0.2)
NEN	1 (0.2)
No pathologic analysis	
Contrast-enhanced CT or MRI	
HCC	42 (10.4)
Hemangioma	33 (8.2)
Follow-up	
< 50% size increase in 12 months	62 (15.4)
≥50% size increase in 12 months	3 (0.7)

Unless otherwise indicated, data are liver nodules or patients (n=403) and data in parentheses are percentages. Mean data are ± standard deviation. HBV, hepatitis B virus; HCV, hepatitis C virus; PBC, primary biliary cirrhosis; HCC, hepatocellular carcinoma; DN, dysplastic nodule; RN, regenerative nodule; FNH, focal nodular hyperplasia; ICC, intrahepatic cholangiocarcinoma; cHCC-CCA, combined hepatocellular-cholangiocarcinoma; NEN, neuroendocrine neoplasm.

^*^Data in parentheses are range.

### Intra- and inter-LLM Agreement on CEUS LI-RADS categorization for small FLLs

The distributions of intra-LLM agreement and inter-LLM agreement are presented in **Table 2**. ChatGPT-4.0 and ChatGPT-4o demonstrated substantial to almost perfect intra-agreement in the CEUS LI-RADS classification assignment among radiologists with varying levels of liver CEUS experience (κ value = 0.76-1[95% CI: 0.69, 1], and 0.7-0.94 [95% CI: 0.55, 0.99] for ChatGPT-4.0, and ChatGPT 4o, respectively). There was moderate to substantial intra-agreement for ChatGPT-4o mini, with κ values ranging from 0.51 to 0.72 (95% CI: 0.33 to 0.79). However, apart from moderate agreement for a junior radiologist, Google Gemini demonstrated poor to fair intra-LLM agreement for the other radiologists (κ value = -0.04 to 0.47 [95% CI: -0.32, 0.6]).

**Table 2:** Intra-LLMs and Inter-LLMs agreements for CEUS LI-RADS category assignments.

Agreement evaluation	Human-LLM Interaction					
	Junior Radiologist		Senior Radiologist		Expert Radiologist	
	1	2	1	2	1	2
Intra-LLM agreement^*^						
Gemini	-0.04 (-0.32-0.5)	0.47 (0.36-0.6)	0.3 (0.15-0.46)	-0.03 (-0.1-0.1)	0.26 (0.12-0.41)	0.07 (-0.1-0.28)
GPT-4o mini	0.71 (0.35, 0.93)	0.72 (0.65, 0.79)	0.68 (0.56, 0.77)	0.59 (0.49, 0.69)	0.62 (0.5, 0.72)	0.51 (0.33, 0.67)
GPT-4.0	1 (1-1)	0.84 (0.8-0.88)	0.88 (0.83-0.9)	0.76 (0.69-0.83)	0.9 (0.86-0.93)	0.95 (0.92-0.97)
GPT-4o	0.94 (0.82-0.99)	0.78 (0.7-0.84)	0.73 (0.63-0.8)	0.8 (0.74-0.85)	0.85 (0.79-0.9)	0.7 (0.55-0.81)
*Inter-LLM agreement^†^*						
Gemini vs GPT-4o mini	-0.14 (-0.7, 0.6)	0.55 (0.41, 0.67)	0.13 (-0.1, 0.36)	-0.3 (-0.47, -0.1)	-0.05 (-0.3, 0.2)	-0.14 (-0.4, 0.2)
Gemini vs GPT-4.0	0.01 (-0.63-0.67)	0.27 (0.09-0.4)	0.06 (-0.18-0.3)	-0.2 (-0.4-0.02)	0.14 (-0.1-0.35)	0.15 (-0.16-0.44)
Gemini vs GPT-4o	0.04 (-0.61-0.68)	0.18 (-0.01-0.4)	0.23 (-0.01-0.4)	-0.05 (-0.2-0.2)	0.04 (-0.2-0.27)	0.16 (-0.15-0.44)
GPT-4o mini vs GPT-4.0	0.41 (-0.3, 0.84)	0.35 (0.18, 0.5)	0.55 (0.36, 0.69)	0.48 (0.32, 0.62)	0.27 (0.05, 0.47)	0.34 (0.04, 0.58)
GPT-4o mini vs GPT-4o	0.66 (0.04, 0.92)	0.19 (-0.01-0.4)	0.47 (0.26, 0.63)	0.34 (0.16, 0.5)	0.25 (0.02, 0.45)	0.16 (-0.15, 0.4)
GPT-4.0 vs GPT-4o	0.86 (0.50-0.97)	0.7 (0.58-0.78)	0.83 (0.7-0.89)	0.71 (0.6-0.8)	0.69 (0.55-0.79)	0.63 (0.4-0.78)

Three levels of radiologists individually generated 403 CEUS LI-RSDS reports, along with a prompt output of a CEUS LI-RADS category. Data are κ values, and data in parentheses are 95% CIs. LLM, large language model; CEUS LI-RADS, contrast-enhanced US Liver Imaging Reporting and Data System.

^*^Kappa values calculated as described by Fleiss’ κ for the Intra-LLM agreement and their 95% CIs.

^†^Kappa values calculated as described by Cohen’ κ for the Inter-LLM agreement and their 95% CIs.

As for the inter-LLM agreement evaluation, GPT-4.0 and GPT-4o achieved substantial to almost perfect agreement for both the junior and senior radiologists (κ value = 0.7-0.86 [95% CI: 0.58, 0.97]), and substantial agreement for the expert radiologists (κ value = 0.63-0.69 [95% CI: 0.4, 0.79]), respectively. ChatGPT-4o mini showed fair to moderate agreement with ChatGPT-4.0 (κ value = 0.27-0.55 [95% CI: 0.05, 0.69]) involving all readers, whereas poor to substantial agreement with ChatGPT-4o (κ value = 0.16-0.66 [95% CI: -0.15, 0.92]). There was poor to fair agreement between Google Gemini and ChatGPT, including version 4o mini, 4.0 and 4o, with κ values ranging from -0.3 to 0.55 (95% CI: -0.47 to 0.67), regardless of the radiologist’s expertise.

### Diagnostic efficacy of ChatGPT-4.0 and ChatGPT-4o in predicting small HCC

The diagnostic performance of LLMs in diagnosing sHCC is shown in **Table 3** and **Supplementary Table S2**. Since ChatGPT-4.0 showed comparable intra-LLM and superior inter-LLM agreement to other LLMs, its diagnostic performance was evaluated in greater detail. In a human-LLM interaction context, ChatGPT-4.0 demonstrated superior sensitivity compared to ChatGPT-4o across all readers levels, achieving 83% [95% CI: 73%, 90%] versus 70% [95% CI: 59%, 79%] for junior radiologists (*p* = 0.007), 86% [95% CI: 78%, 92%] versus 77% [95% CI: 68%, 84%] for senior radiologists (*p* = 0.02), and 90% [95% CI: 81%, 95%] versus 78% [95% CI: 67%, 87%] for expert radiologists (*p* = 0.004), respectively. However, ChatGPT-4.0 and ChatGPT-4o exhibited comparable specificity in differentiating sHCC from non-HCC. Regarding diagnostic accuracy, ChatGPT-4.0 demonstrated superior performance compared to ChatGPT 4o for senior (87% [95% CI: 81%, 92%] vs 79% [95% CI: 72%, 85%], *p* = 0.009) and expert radiologists (90% [95% CI: 83%, 94%] vs 80% [95% CI: 72%, 87%], *p* = 0.001). However, they showed comparable performance for junior radiologists (81% [95% CI: 73%, 88%] vs 74% [95% CI: 65%, 82%], *p* = 0.12). Similarly, the AUC for ChatGPT-4.0 was higher for senior and expert radiologists (*p* = 0.01 and *p* = .001, respectively), but comparable for junior radiologists, when compared with ChatGPT-4o.

**Table 3:** Comparison of ChatGPT 4.0 and ChatGPT 4o in predicting small HCC versus Non-HCC.

Diagnostic Performance	Human-LLM Interaction		
	Junior Radiologist	Senior Radiologist	Expert Radiologist
Sensitivity (%)			
GPT-4.0	83 (72/87)	86 (93/108)	89 (69/77)
GPT-4o	70 (61/87)	77 (83/108)	78 (60/77)
*p* Value	0.007	0.02	0.004
Specificity (%)			
GPT-4.0	76 (22/29)	89 (56/63)	90 (35/39)
GPT-4o	86 (25/29)	83 (52/63)	85 (33/39)
*p* Value	0.38	0.34	0.50
Accuracy (%)			
GPT-4.0	81(94/116)	87 (149/171)	90 (104/116)
GPT-4o	74 (86/116)	79 (135/171)	80 (93/116)
*p* Value	0.12	0.009	0.001
AUC			
GPT-4.0	0.79 (0.71, 0.86)	0.88 (0.82, 0.92)	0.89 (0.83, 0.95)
GPT-4o	0.78 (0.70, 0.85)	0.79 (0.73, 0.86)	0.81 (0.73, 0.88)
*p* Value	0.79	0.01	0.001

Data in parentheses for the sensitivity, specificity and accuracy are numerator/denominator; data in parentheses for the AUC are 95% confidence intervals. *p* values present the comparison of performance between ChatGPT 4.0 and ChatGPT 4o in predicting small HCC using the same input generated by the same radiologist. LLM, large language model; HCC, hepatocellular carcinoma, AUC = area under a receiver operating characteristic curve.

### Performance of human-LLM interaction, CEUS LI-RADS and CNN strategy in diagnosing small HCC

**Figure 4:**
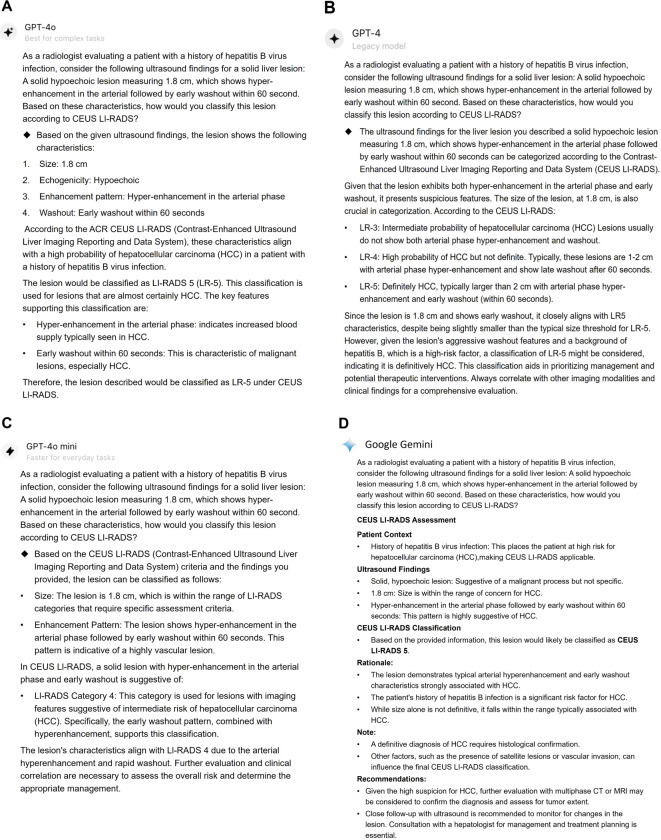
Responses for a CEUS LI-RADS LR-M category small liver lesion classified by LLMs. This lesion was classified as CEUS LI-RADS LR-5 category by ChatGPT-4o **(A)**, ChatGPT-4.0 **(B)**, Google Gemini **(D)**, however, it was assigned to LR-4 by ChatGPT-4o mini **(C)**. The lesion was confirmed as a moderately-differentiated HCC by histopathology. CEUS LI-RADS, Contrast-enhanced Ultrasound Liver Imaging Reporting and Data System; LLMs, large language models; HCC, hepatocellular carcinoma.

**Figure 5:**
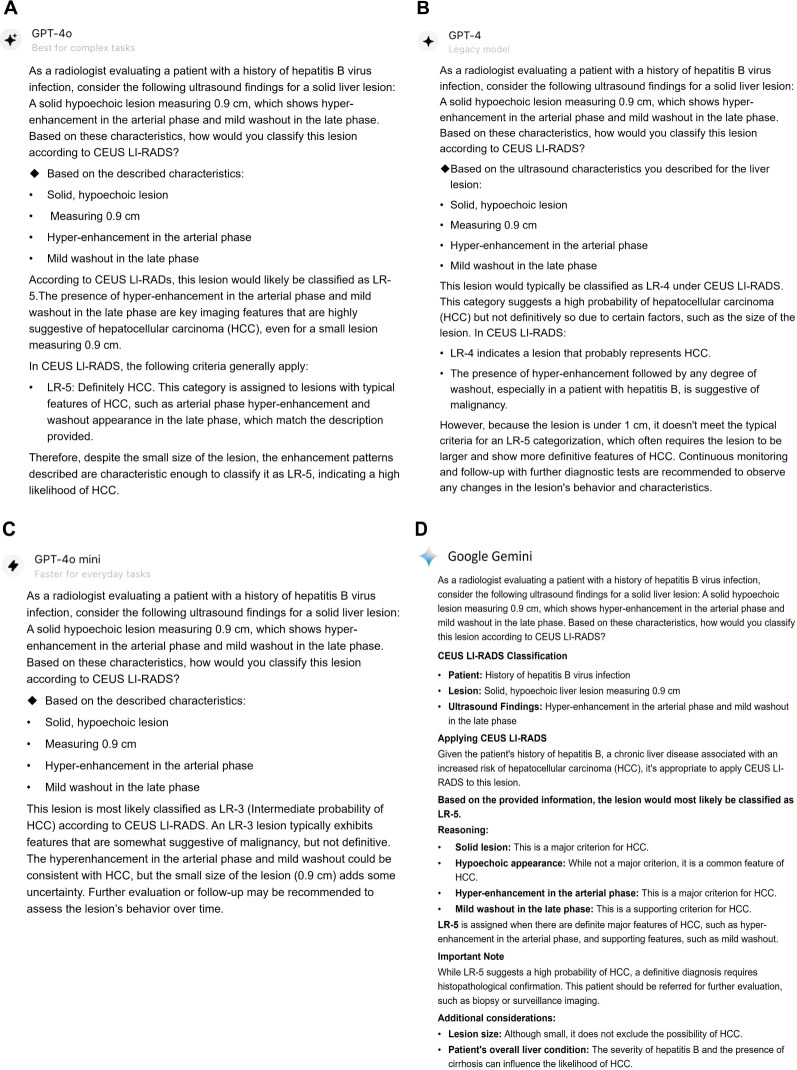
Responses for a CEUS LI-RADS LR-4 category small liver lesion classified by LLMs. The lesion was classified as CEUS LI-RADS LR-5 category by ChatGPT-4o **(A)** and Google Gemini **(D)**. However, it was categorized as LR-4 and LR-3 by ChatGPT-4.0 **(B)** and ChatGPT-4o mini, respectively. The lesion was confirmed as a poorly-differentiated HCC by histopathology. CEUS LI-RADS, Contrast-enhanced Ultrasound Liver Imaging Reporting and Data System; LLMs, large language models; HCC, hepatocellular carcinoma.

**Table 4** shows the diagnostic effectiveness for sHCC by ChatGPT-4.0, human readers using CEUS LI-RADS, and a CNN strategy. ChatGPT-4.0 achieved significantly higher sensitivities of 83% (95% CI: 73%, 90%), 86% (95% CI: 78%, 92%), and 89% (95% CI: 81%, 95%) for junior, senior, and expert radiologists, respectively, compared to human readers with corresponding liver CEUS expertise, who demonstrated sensitivities of 63% (95% CI: 52%, 73%) (*p* <.001), 69% (95% CI: 60%, 78%) (*p* < 0.001), and 78% (95% CI: 67%, 86%) (*p* = 0.004). Besides, ChatGPT-4.0 had similar specificity (76%-90% [95% CI: 56%, 97%] vs 90%-95% [95% CI: 73%, 99%]) to that of human readers, with all P-values above.05. As for accuracy, ChatGPT-4.0 achieved 81% (95% CI: 73%, 88%) for the junior radiologist and 87% (95% CI: 81%, 91%) for the senior radiologist, outperforming human readers who showed accuracies of 70% (95% CI: 61%, 78%) for the junior radiologist (*p* = 0.007) and 79% (95% CI: 72%, 85%) for the senior radiologist (*p* = 0.004), respectively. Notably, ChatGPT-4.0 showed comparable accuracy to that of the expert radiologist (90% [95% CI: 83%, 94%] vs 84% [95% CI: 76%, 90%], *p* =0.07) and AUC (0.89 [95% CI: 0.83, 0.95] vs 0.86 [95% CI: 0.79, 0.92], *p* = 0.20). Examples of LLMs for the CEUS LI-RADS category for sHCC are presented in **Figures 4** and **5**.

**Table 4:** Diagnostic performance of ChatGPT-4.0, human reader, and US Images-based CNN model in predicting small HCC versus Non-HCC.

Diagnostic Performance	SEN (%)	*p* Value	SPE (%)	*p* Value	ACC (%)	*p* Value	AUC^‡^	*p* Value
*ChatGPT-4.0 vs Human Reader*								
ChatGPT-4.0								
Junior Radiologist	83 (72/87)	<0.001^*^	76 (22/29)	0.13^*^	81 (94/116)	0.007^*^	0.79 (0.71, 0.86)	0.46^*^
Senior Radiologist	86 (93/108)	<0.001^*^	89 (56/63)	0.13^*^	87 (149/171)	0.004^*^	0.88 (0.82, 0.92)	0.03^*^
Expert Radiologist	89 (69/77)	0.004^*^	90 (35/39)	0.50^*^	90 (104/116)	0.07^*^	0.89 (0.83, 0.95)	0.20^*^
Human Reader with CEUS LI-RADS								
Junior Radiologist	63 (55/87)		90 (26/29)		70 (81/116)		0.76 (0.68, 0.84)	
Senior Radiologist	69 (75/108)		95 (60/63)		79 (135/171)		0.82 (0.76, 0.88)	
Expert Radiologist	78 (60/77)		95 (60/77)		84 (97/116)		0.86 (0.79, 0.92)	
*ChatGPT-4.0 vs CNN*								
ChatGPT-4.0	86 (94/110)	0.004^†^	87 (45/52)	<0.001^†^	86 (139/162)	0.01^†^	0.86 (0.8, 0.91)	<0.001^†^
CNN	96 (106/110)		29 (15/52)		75 (121/162)		0.63 (0.55, 0.7)	

Unless otherwise indicated, data in parentheses are numerators/denominators. CNN, convolutional neural network; HCC, hepatocellular carcinoma; SEN, sensitivity; SPE, specificity; ACC, accuracy; AUC, area under a receiver operating characteristic curve.

^*^ *p* values present the comparison of performance between ChatGPT-4.0 and the human reader who generated the original structured CEUS LI-RADS reports.

^†^ *p*values are for comparing the diagnostic performance between ChatGPT-4.0 and CNN model.

^‡^Data in parentheses are 95% confidence intervals.

The CNN model showed higher sensitivity at 96% (95% CI: 91%, 97%) compared to 86% (95% CI: 77%, 91%) for ChatGPT-4.0 with CEUS LI-RADS (*p* = 0.004), but lower specificity at 29% [95% CI: 82%, 87%] versus 87% [95% CI: 74%, 94%] (*p =* < 0.001). Moreover, ChatGPT-4.0 exhibited superior accuracy and AUC compared to the CNN model, with an accuracy of 86% (95% CI: 79%, 91%) versus 75% (95% CI: 67%, 81%, *p* = 0.01), and an AUC of 0.86 (95% CI: 0.80, 0.91) versus 0.63 (95% CI: 0.55, 0.70, *p* < 0.001).

Additionally, the diagnostic performance of ChatGPT-4.0, human readers using CEUS LI-RADS, and a CNN model for malignant sFLLs was investigated, as shown in **Supplementary Table S3**. **Supplementary Table S4** presents the performance of ChatGPT-4o, ChatGPT-4o mini and Genimi in differentiating malignant from benign sFLLs.

## Discussion

In this study, we investigated the intra- and inter-agreement, as well as the diagnostic accuracy of four popular large language models (LLMs) in diagnosing small hepatocellular carcinoma (sHCC) in high-risk patients. ChatGPT-4.0 and ChatGPT-4o showed substantial to almost perfect intra-agreement (κ = 0.76-1 and 0.7-0.94, respectively) and higher inter-agreement than other LLMs (κ = 0.63-0.86). In human-LLM interactions using CEUS LI-RADS, ChatGPT-4.0 demonstrated comparable specificity (76%-90%) across radiologists with varying levels of liver CEUS expertise, similar to ChatGPT-4o (83%-86%). However, ChatGPT-4.0 outperformed ChatGPT-4o with a sensitivity of 83%-89% versus 70%-78%, *p ≤* 0.02. Notably, ChatGPT-4.0 demonstrated superior sensitivity, ranging from 83% to 89% compared to 63% to 78% for human readers (*p* ≤ 0.004), in diagnosing sHCC. Moreover, ChatGPT-4.0 with CEUS LI-RADS outperformed CNN models in predicting sHCC with AUC of 0.86 versus 0.63 (*p* < 0.001). Overall, ChatGPT-4o mini and Google Gemini showed poor intra- and inter-LLM agreement and lower diagnostic efficacy in diagnosing sHCC compared to ChatGPT-4.0 and ChatGPT-4o.

Currently, the primary focus of LLMs in the diagnostic imaging field is on processing text data, though research is now extending these models to multimodal tasks (such as combining image and text processing) (20, 21). The CEUS LI-RADS released by ACR provides a diagnostic framework for assessing the risk of HCC in patients at risk. However, imaging early-stage HCC, particularly lesions under 2 cm is challenging (22). We previously determined that CEUS LI-RADS effectively characterizes sFLLs (12), while the interaction between LLMs and CEUS LI-RADS in diagnosing liver nodules, especially sFLLs, remains unexplored. By removing spatiotemporal interference factors, we found that ChatGPT-4.0 and ChatGPT-4o achieved superior repeatability in CEUS LI-RADS categorization among the four LLMs. GPT-4o mini is characterized by faster processing and superior intelligence compared to ChatGPT-3.5. However, similar to Google Gemini, it demonstrates poor reproducibility in CEUS LI-RADS classification. This is of considerable significance because the stable and reliable grasp of the CEUS LI-RADS system by LLMs could potentially establish a foundation for their clinical diagnostic applications.

Notably, human-LLM interaction with ChatGPT-4.0 outperformed the radiologists who generated the original structured CEUS LI-RADS reports in sensitivity and accuracy in diagnosis sHCC. Interestingly, we observed that, 15.8% (43 of 272) of sHCC were classified as LR-M by human readers, and of these, 88.4% (38 of 43) were assigned to LR-5 by ChatGPT-4.0. Previous studies have shown that early washout within 60 seconds—an essential LR-M feature—is the main factor causing many HCC cases to be classified as LR-M (23–25). In the study by Zheng et al, the investigators found that of 354 LR-M nodules, 224 (63%) were HCC (23). By recategorizing nodules displaying early washout and without punched-out before 5 minutes into LR-5, the sensitivity could elevate from 75% (1141 of 1513) to 85% (1283 of 1513) (P <.001), and accuracy from 81% to 87% (P <.001). Although we have demonstrated that ChatGPT-4.0 understands the CEUS LI-RADS system and recognizes early washout as a typical feature of LR-M, it continues to classify nodules showing hyper-enhancement in arterial phase followed by early washout as LR-5. ChatGPT-4.0 seems to incorporate recent research and does not strictly classify a case as LR-M if washout occurs within 60 seconds. This could be the core reason why ChatGPT-4.0 demonstrated higher sensitivity compared to human readers using CEUS LI-RADS criteria. Considering the “black box” nature of LLMs, ongoing efforts are essential to address issues such as clearly explaining how decisions are derived. The aforementioned finding highlights the valuable role of LLMs in enhancing the accuracy of diagnoses made by radiologists, not only for senior ultrasound practitioners but also for senior practitioners to make more comprehensive judgements.

Despite the innovative application of LLM in diagnosing sHCC with CEUS LI-RADS, our study has some limitations. First, the LLM task relied only on structured CEUS LI-RADS reports, limiting access to full imaging and clinical information about the patients. Second, the sample sizes for certain CEUS LI-RADS categories, especially LR-2, were small, likely due to the infrequent use of CEUS for liver nodules under 1 cm in routine practice. Third, our study focused on ≤2 cm sFLLs in high-risk patients, which limited the number of participants, and the lack of sufficient follow-up led to additional exclusions. The COVID-19 pandemic and related control measures further reduced patient enrollment, with only 5, 11, and 15 patients being included in 2020, 2021, and 2022, respectively.

In conclusion, large language models (LLMs) showed significant potential in diagnosing small hepatocellular carcinoma (sHCC) in high-risk patients when integrated with the CEUS LI-RADS. ChatGPT-4.0 and ChatGPT-4o demonstrated satisfactory reproducibility with ChatGPT-4.0 outperforming ChatGPT-4o, ChatGPT-4o Mini, and Google Gemini in diagnostic efficacy. It is worth noting that ChatGPT-4.0 identified the ‘early washout’ feature would not rule out LR-5, which may be the core reason for its superior sensitivity and accuracy in detecting sHCC compared to human readers using CEUS LI-RADS. This highlights the need for continuous data review, model refinement, and improved transparency and explainability of LLM decision-making.

## Data Availability

The original contributions presented in the study are included in the article/**Supplementary Material**. Further inquiries can be directed to the corresponding authors.
