# Supplementary material for: Feasibility of large language models for CEUS LI-RADS categorization of small liver nodules in patients at risk for hepatocellular carcinoma
Source: Front Oncol. 2024 Dec 18;14:1513608. doi: 10.3389/fonc.2024.1513608 (PMC11688206; doi:10.3389/fonc.2024.1513608)
Supplement: Supplementary file 1 [file Table1.docx]

**Supplementary Material**

**Appendix S1**

**CEUS Examination Processing**

Baseline ultrasound and CEUS examinations were performed using EPIQ7 and IU 22 (Philips Healthcare) both equipped with C5-1 probes or Mindray Resona 7 ultrasound system (Mindray Medical Solutions) with an SC6-1 transducer. The CEUS examination was conducted with SonoVue (Bracco) in accordance with the manufacturer’s instructions, with a bolus injection of 1.2–2.4 mL administered via a 20-gauge catheter inserted into the antecubital vein. Both still images and video clips from the B-mode and CEUS examinations were digitally stored for further evaluation. Six readers who were blinded to reference standard, independently reviewed ultrasound data and recorded the imaging characteristics in Excel spreadsheets.

**Appendix S2**

**Generation of Structured Reports**

The US imaging data of the patients were processed as individual files and randomly numbered after de-identification by a radiologist who was not among the readers reviewing the US examinations. A preliminary Excel spreadsheet with patients’ numerals, age, sex, and the location of small FLLs was provided to six radiologists with varying liver CEUS expertise. Among them, two were junior radiologists (K.Y.Z., and R.Y.) with 3-5 years of liver CEUS expertise, two were senior radiologists (J.L., and J.Y.H.) with 5-8 years of liver CEUS expertise, and two were expert practitioners (Q.L., and Y.L.) with over 10 years of professional experience in this field. The US imaging characteristic of included sFLLs were recorded into the aforementioned Excel spreadsheet by each reader after independently analysis. The following diagnostic features were used to characterize each lesion based on CEUS LI-RADS: lesion size; arterial phase enhancement and its pattern; presence, timing, and degree of washout; tumor in vein, mosaic and nodule-in-nodule architecture; and size change at follow-up imaging^[1]^. Structured CEUS reports were generated based on data from the Excel spreadsheet.


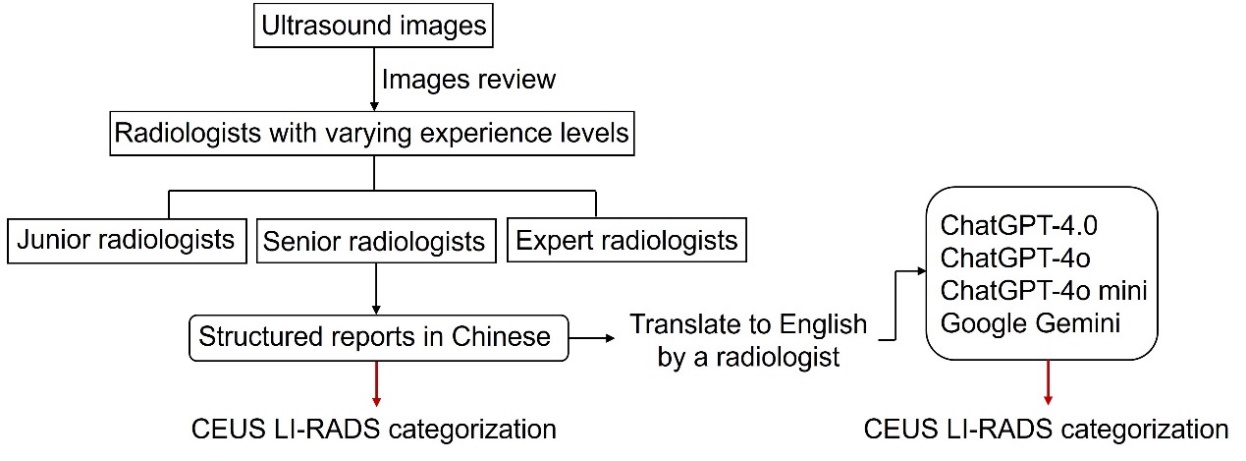


**Figure S1:** Schematic diagram of how human readers and LLMs render CEUS LI-RADS categorization.

**Appendix S3**

**End-to-End Convolutional Neural Network (CNN) Model development**

The end-to-end CNN models for differentiating small HCC from non-HCC, and small malignant from benign FLLs was developed as previously described^[2]^. In brief, the ResNet-50 architecture was utilized to train on 964 ultrasound images, encompassing both B-mode and CEUS images (Table S1). The images from B-mode and CEUS-AP, PP, and LP were trained separately, with an ensemble output derived through voting calculations. The final weighting ratio for network branches was determined using ultrasound images from B-mode and multiple phases of CEUS: 0.2 for B-mode, 0.4 for AP, 0.2 for PP, and 0.2 for LP (Fig S2).


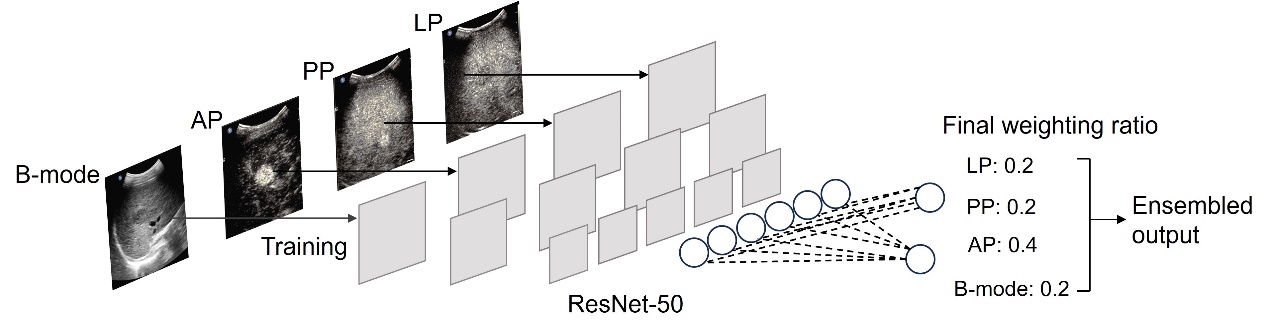


**Figure S2:** Schematic diagram of CNN model construction. AP = arterial phase, PP = portal phase, LP = late phase, ResNet = Residual Network.

| **Table S1: Network Structures and Parameters** | | |
| --- | --- | --- |
| Stage | Output | ResNet-50 |
| Dropout | \ | Yes |
| Other regularization | \ | Batch Normalization |
| Stem | 112 × 112 | 7 × 7, 64, stride 2 |
|  | 56 × 56 | 3 × 3 max pool, stride 2 |
| Block 1 | 56 × 56 | 1×1, 64  3×3, 64  1×1, 256  × 3 |
| Block 2 | 28 × 28 | 1×1, 128  3×3, 128  1×1, 512  × 4 |
| Block 3 | 14 × 14 | 1×1, 256  3×3, 256  1×1, 1024  × 6 |
| Block 4 | 7 × 7 | 1×1, 512  3×3, 512  1×1, 2048  × 3 |
|  | 1 × 1 | Average pool, softmax |

| **Table S2:** **Diagnostic performance of ChatGPT-4o mini and Genimi in Predicting Small HCC versus non-HCC** | | | |
| --- | --- | --- | --- |
| Diagnostic Performance | Human-LLM Interaction | | |
|  | Junior Radiologist | Senior Radiologist | Expert Radiologist |
| Sensitivity (%) |  |  |  |
| GPT-4o mini | 17 (15/87) | 12 (13/108) | 9 (7/77) |
| Genimi | 10 (9/87) | 9 (10/108) | 4 (3/77) |
| Specificity (%) |  |  |  |
| GPT-4o mini | 76 (22/29) | 92 (58/63) | 92 (36/39) |
| Genimi | 90 (26/29) | 97 (61/63) | 92 (36/39) |
| Accuracy (%) |  |  |  |
| GPT-4o mini | 32 (37/116) | 41 (71/171) | 37 (43/116) |
| Genimi | 30 (25/116) | 41 (71/171) | 34 (39/116) |
| AUC |  |  |  |
| GPT-4o mini | 0.47 (0.37, 0.56) | 0.52 (0.44, 0.60) | 0.51 (0.41, 0.6) |
| Genimi | 0.50(0.41, 0.59) | 0.53 (0.45, 0.61) | 0.48 (0.39, 0.58) |
| Note.—Data in parentheses for the sensitivity, specificity and accuracy are numerator/denominator; data in parentheses for the AUC are 95% confidence intervals. HCC = hepatocellular carcinoma, AUC = area under a receiver operating characteristic curve. | | | |

| **Table S3: Diagnostic Performance of ChatGPT-4.0, human reader, and US Images-based CNN Model in Predicting Small Benign versus Malignant FLLs** | | | | | | | | | | | |
| --- | --- | --- | --- | --- | --- | --- | --- | --- | --- | --- | --- |
| Diagnostic Performance | SEN (%) | *p*  Value | | SPE (%) | | *p*  Value | ACC (%) | | *p*  Value | AUC^‡^ | *p*  Value |
| ***ChatGPT-4.0 vs Human Reader*** | | |  |  |  | |  | |  |  |  |
| ChatGPT-4.0 |  | |  |  |  | |  |  | |  |  |
| Junior Radiologist | 98 (89/91) | | 0.99^*^ | 64 (16/25) | 0.68^*^ | | 91 (105/116) | | 0.45^*^ | 0.81 (0.73, 0.88) | 0.42^*^ |
| Senior Radiologist | 97 (110/114) | | 0.69^*^ | 77 (44/57) | 0.99^*^ | | 91 (154/171) | | 0.99^*^ | 0.87 (0.81, 0.92) | 0.35^*^ |
| Expert Radiologist | 96 (77/80) | | 0.99^*^ | 67 (24/36) | 0.13^*^ | | 87 (101/116) | | 0.18^*^ | 0.82 (0.73, 0.88) | 0.08^*^ |
| Human Reader |  | |  |  |  | |  | |  |  |  |
| Junior Radiologist | 98 (89/91) | |  | 72 (18/25) |  | | 92 (107/116) | |  | 0.85 (0.77, 0.91) |  |
| Senior Radiologist | 98 (112/114) | |  | 79 (45/57) |  | | 92 (157/171) | |  | 0.89 (0.83, 0.93) |  |
| Expert Radiologist | 95 (76/80) | |  | 80 (29/36) |  | | 91 (105/116) | |  | 0.88 (0.8, 0.93) |  |
| ***ChatGPT-4.0 vs CNN*** | | |  |  |  | |  | |  |  |  |
| ChatGPT-4.0 | 96 (112/116) | | 0.99^†^ | 72 (33/46) | <0.001^†^ | | 90 (145/162) | | <0.001^†^ | 0.84 (0.78, 0.89) | <0.001^†^ |
| CNN | 97 (113/116) | |  | 22 (10/46) |  | | 76 (123/162) | |  | 0.60 (0.52, 0.67) |  |
| Note.—The indication of malignancy included categories LR-M, LR-5, and LR-4, whereas LR-3, LR-2, and LR-1 were indicative of benign FLLs. CNN = convolutional neural network, FLL = focal liver lesions, SEN = sensitivity, SPE = specificity, ACC = accuracy, AUC = area under a receiver operating characteristic curve.  ^*^ *P* values present the comparison of performance between ChatGPT-4.0 and the human reader who generated the original structured CEUS LI-RADS reports.  ^†^ *P* values are for comparing the diagnostic performance between ChatGPT-4.0 and CNN model.  ^‡^ Data in parentheses are 95% confidence intervals. | | | | | | | | | | | |

| **Table S4: Diagnostic** **performance of ChatGPT-4o, ChatGPT-4o mini and Genimi in Differentiating Small malignant from benign FLLs** | | | |
| --- | --- | --- | --- |
| Diagnostic Performance | Human-LLM Interaction | | |
|  | Junior Radiologist | Senior Radiologist | Expert Radiologist |
| Sensitivity (%) |  |  |  |
| GPT-4o | 98 (90/91) | 96 (110/114) | 96 (77/80) |
| GPT-4o mini | 86 (78/91) | 83 (95/114) | 81 (65/80) |
| Genimi | 91 (83/91) | 92 (105/114) | 95 (76/80) |
| Specificity (%) |  |  |  |
| GPT-4o | 56 (14/25) | 60 (34/57) | 42 (15/36) |
| GPT-4o mini | 56 (14/25) | 70 (40/57) | 58 (21/36) |
| Genimi | 28 (7/25) | 21 (12/57) | 19 (7/36)) |
| Accuracy (%) |  |  |  |
| GPT-4o | 89 (104/116) | 84 (144/171) | 79 (92/116) |
| GPT-4o mini | 79 (92/116) | 79 (135/171) | 74 (76/116) |
| Genimi | 77 (90/116) | 68 (117/171) | 71 (83/116) |
| AUC |  |  |  |
| GPT-4o | 0.77 (0.69, 0.85) | 0.78 (0.71, 0.84) | 0.69 (0.60, 0.77) |
| GPT-4o mini | 0.71 (0.62, 0.79) | 0.77 (0.70, 0.83) | 0.70 (0.61, 0.78) |
| Genimi | 0.60 (0.50, 0.69) | 0.57 (0.49, 0.64) | 0.57 (0.48, 0.67) |
| Note.—Data in parentheses for the sensitivity, specificity and accuracy are numerator/denominator; data in parentheses for the AUC are 95% confidence intervals. HCC = hepatocellular carcinoma, AUC = area under a receiver operating characteristic curve. | | | |

**References**

[1] Lyshchik A, Kono Y, Dietrich CF, et al. Contrast-enhanced ultrasound of the liver: technical and lexicon recommendations from the ACR CEUS LI-RADS working group. Abdom Radiol (NY). 2018. 43(4): 861-879.

[2] Wu SH, Tong WJ, Li MD, et al. Collaborative Enhancement of Consistency and Accuracy in US Diagnosis of Thyroid Nodules Using Large Language Models. Radiology. 2024. 310(3): e232255.
